# Severe endothelial injury and subsequent repair in patients after successful cardiopulmonary resuscitation

**DOI:** 10.1186/cc9050

**Published:** 2010-06-04

**Authors:** Katrin Fink, Meike Schwarz, Linda Feldbrügge, Julia N Sunkomat, Tilmann Schwab, Natascha Bourgeois, Manfred Olschewski, Constantin von zur Mühlen, Christoph Bode, Hans-Jörg Busch

**Affiliations:** 1 Department of Cardiology and Angiology, University hospital of Freiburg, Hugstetter Str. 55, 79106 Freiburg im Breisgau, Germany; 2 Department for Biometry and Statistics, University of Freiburg, Stefan-Meier-Str. 26, 79104 Freiburg im Breisgau, Germany

## Abstract

**Introduction:**

Ischemia and reperfusion after cardiopulmonary resuscitation (CPR) induce endothelial activation and systemic inflammatory response, resulting in post-resuscitation disease. In this study we analyzed direct markers of endothelial injury, circulating endothelial cells (CECs) and endothelial microparticles (EMPs), and endothelial progenitor cells (EPCs) as a marker of endothelial repair in patients after CPR.

**Methods:**

First we investigated endothelial injury in 40 patients after CPR, 30 controls with stable coronary artery disease (CAD), and 9 healthy subjects, who were included to measure CECs and EMPs. In a subsequent study, endothelial repair was assessed by EPC measurement in 15 CPR, 9 CAD, and 5 healthy subjects. Blood samples were drawn immediately and 24 hours after ROSC and analyzed by flow cytometry. For all statistical analyses *P *< 0.05 was considered significant.

**Results:**

There was a massive rise in CEC count in resuscitated patients compared to CAD (4,494.1 ± 1,246 versus 312.7 ± 41 cells/mL; *P *< 0.001) and healthy patients (47.5 ± 3.7 cells/mL; *P *< 0.0005). Patients after prolonged CPR (≥30 min) showed elevated CECs compared to those resuscitated for <30 min (6,216.6 ± 2,057 versus 2,340.9 ± 703.5 cells/mL; *P *= 0.13/ns). There was a significant positive correlation of CEC count with duration of CPR (R^2^= 0.84; *P *< 0.01). EMPs were higher immediately after CPR compared to controls (31.2 ± 5.8 versus 19.7 ± 2.4 events/μL; *P *= 0.12 (CAD); versus 15.0 ± 5.2 events/μL; *P *= 0.07 (healthy)) but did not reach significance until 24 hours after CPR (69.1 ± 12.4 versus 22.0 ± 3.0 events/μL; *P *< 0.005 (CAD); versus 15.4 ± 4.4 events/μL; *P *< 0.001 (healthy)). EPCs were significantly elevated in patients on the second day after CPR compared to CAD (1.16 ± 0.41 versus 0.02 ± 0.01% of lymphocytes; *P *< 0.005) and healthy (0.04 ± 0.01; *P *< 0.005).

**Conclusions:**

In the present study we provide evidence for a severe endothelial damage after successful CPR. Our results point to an ongoing process of endothelial injury, paralleled by a subsequent endothelial regeneration 24 hours after resuscitation.

## Introduction

The clinical course of patients after successful cardiopulmonary resuscitation (CPR) is often complicated by post-resuscitation disease, a condition of multiple life-threatening disorders related to whole-body ischemia and reperfusion syndrome [1,2]. This phenomenon shares many features with severe sepsis, including a systemic inflammatory response with plasma cytokine elevation, coagulation abnormalities, and myocardial dysfunction [3]. Ischemia, reperfusion and hypoxia during or after CPR induce generalized tissue damage with release of reactive oxygen species and endothelial-leukocyte interaction and activation, resulting in increased microvascular permeability and, hence, in loss of endothelial integrity [2]. Several studies demonstrated an endothelial activation with a consecutive endothelial injury following cardiac arrest [4-6] and in models of ischemia and reperfusion [7,8].

A new tool for evaluation of endothelial injury is detection of circulating endothelial cells (CECs): these cells detach from the intima monolayer in response to endothelial damage and become measurable in peripheral blood. Although CECs are rarely found in the blood of healthy individuals, raised numbers are present in patients with a wide variety of diseases involving the endothelium such as vasculitis [9], arterial occlusive disease [10], and cardiovascular disease [11]. There is a strong relation between CECs and endothelial dysfunction because numbers of CECs correlate with surrogate markers of disturbed endothelial function such as flow-mediated dilation and von Willebrand factor (vWF) [12]. Plasma vWF is one of the most established plasma surrogate markers of endothelial damage or dysfunction [13]. VWF antigen concentrations are elevated on the second day after CPR and seem to be an early predictor of outcome [14].

Additionally, there is an increase of endothelial microparticles (EMPs) in states of disturbed endothelial function. Microparticles are small shed membranous vesicles (<1 μm) that are released from cells upon activation or during apoptosis. They reflect the state of their parental cells in amounts and phenotypes [15]. Microparticles have procoagulant properties, modulate endothelial function, and play a role in inflammatory processes [16]. EMPs were found to be elevated in peripheral blood of patients suffering from acute coronary syndrome [17], or peripheral arterial disease [18]. Circulating endothelial progenitor cells (EPCs) are capable of repairing damaged endothelium and furthermore contribute to re-endothelialization and neovascularization [19]. These cells are bone marrow-derived pluripotent vascular progenitor cells that home in on the sites of ischemia and vascular injury [20]. A decrease in EPC count in peripheral blood is associated with endothelial dysfunction [21]. Patients with coronary artery disease showed reduced levels of EPCs [22] and there was an inverse correlation of number of EPCs in the peripheral blood, increased atherosclerotic risk factors, and a higher cardiovascular morbidity and mortality [23].

In the present study, we hypothesize that endothelial injury takes place during and after CPR, which in turn may contribute to post-resuscitation disease. Therefore, the aim of the present study is to detect direct markers of endothelial damage such as CECs, EMPs and vWF, as well as markers of endothelial repair (EPCs) in peripheral blood of patients after successful CPR.

## Materials and methods

### Patients and blood sampling protocol

After the approval of the ethics committee of our institution for both studies (EK-Freiburg 115/07), we first included 40 patients who underwent CPR after cardiac arrest in a prospective study to measure endothelial injury and compared them with 30 patients suffering from stable coronary artery diseases (CAD) and 9 healthy subjects. Subsequently, we prospectively included 15 resuscitated patients to detect endothelial repair. Nine CAD patients and five healthy subjects served as controls. As elevation of CECs, microparticles and EPCs have been described to increase in various malignancies and in severe sepsis, patients with malignant diseases and sepsis were excluded from the study [24]. Patients younger than 18 years and trauma patients were also excluded. Informed consent was obtained *post hoc *from patients surviving with good neurological outcome, or from their relatives in the case of nonsurviving patients. Informed consent was given by all patients in the control groups.

Using an arterial catheter, blood samples in resuscitated patients were collected immediately after admission to the ICU and a second sample was collected 24 hours after return to spontaneous circulation (ROSC) in the CEC and EMP study. EPC study samples were collected on the second day after ROSC. In control patients, blood was drawn from the arterial catheter immediately after percutaneous coronary intervention (PCI). On the second day after PCI and in controls who did not receive PCI, blood was drawn by venopuncture. Samples were drawn slowly, handled carefully and analyzed immediately after sampling. For vein puncture, we used a 21-gauge butterfly needle and discarded the first 7.5 mL.

Flow cytometric analysis was performed on a three-color flow cytometer (FACSCalibur™, BD Biosciences, San Jose, CA, USA) with individual settings for each antibody utilizing Cell Quest Pro™software (BD Biosciences, San Jose, CA, USA).

### Detection of CECs by flow cytometry analysis

**Figure 1:**
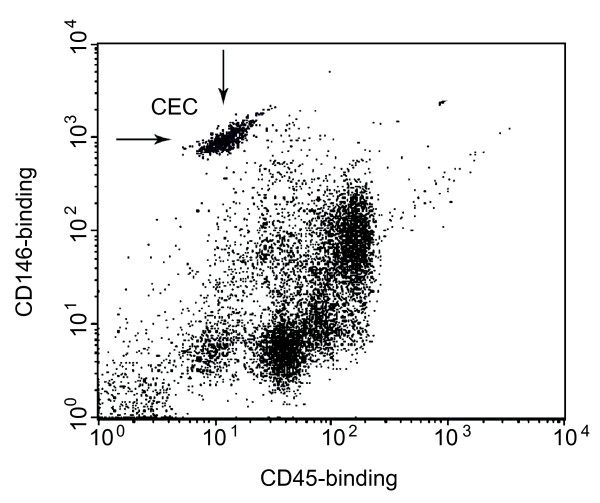
**Flow cytometric detection of circulating endothelial cells in peripheral blood**. Three-color flow cytometry evaluation of circulating endothelial cells (CECs). CD 146-positive and CD 45-negative cells were identified as CECs. In the panel, CEC appear on the upper left as a separate cell population (arrows).

For measurement of CECs, 2.5 mL of blood was collected in EDTA tubes. CECs were detected by a commercially available detection kit (Biocytex, Marseille, France) according to the manufacturer's instructions. CECs were isolated from whole blood by ferromagnetic separation and stained with fluorochrome-labelled monoclonal antibodies (mAb), namely anti-human fluorescein isothiocyanate (FITC)-CD45 and anti-human PE-CD146 for cell detection or anti-human FITC-CD45 and anti-mouse PE-IgG serving as control, respectively. Tubes were analyzed by flow cytometry analysis. Cells larger than the counting beads and with at least the granularity of lymphocytes were gated after identification on the forward/sideward scatter. In this gate, CECs were identified as positive for the specific marker CD146 (melanoma cell adhesion molecule (MCAM), a cell-adhesion molecule used as a marker for endothelial cell lineage) and negative for the hematopoietic marker CD45 (PTPRC, present on all differentiated hematopoietic cells; Figure 1). Samples were analyzed at a flow rate of 60 μl/min for 200 seconds.

### Detection of activated EMPs by flow cytometry analysis

**Figure 2:**
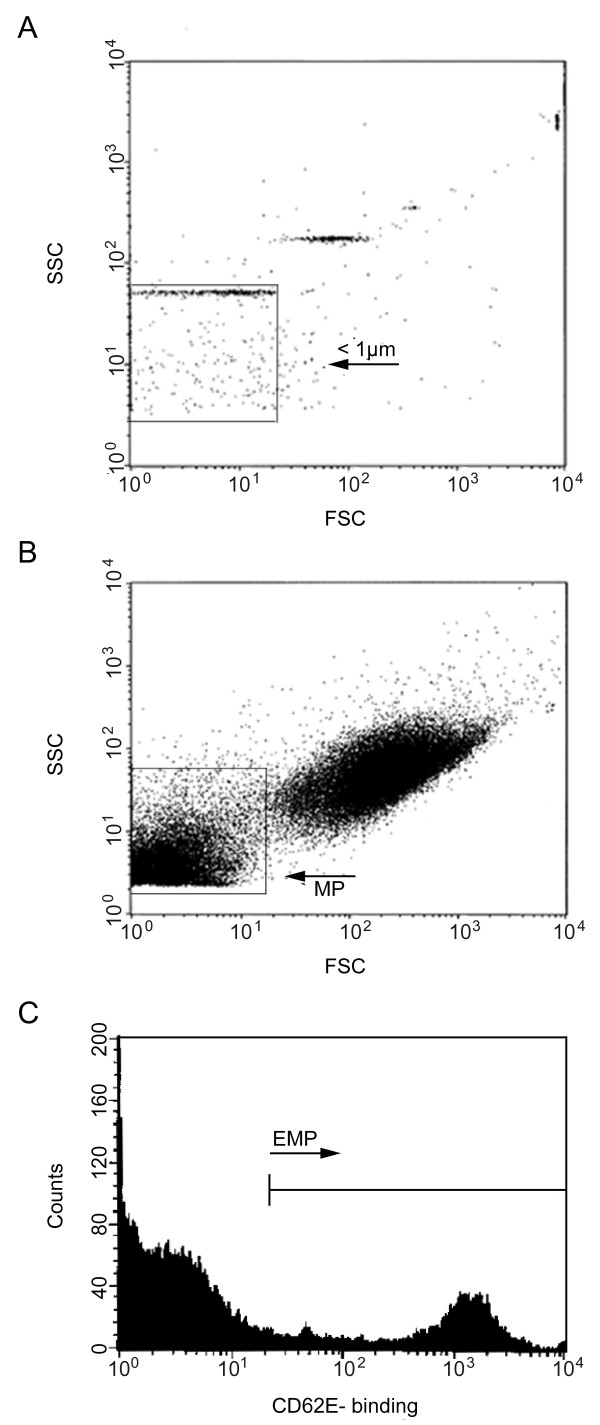
**Flow cytometric detection of endothelial microparticles in peripheral blood**. Three-color flow cytometry evaluation of endothelial microparticles (EMPs). **(a) **Detection of particles with a size of less than 1 μm by nano-beads, **(b) **then gating of microparticles in the lower right. **(c) **Events staining positive for the fluorochrome-labelled antibody directed against E-selectin (CD62E) were identified as EMPs. FSC, forward scatter; SSC, side scatter.

For analysis of EMP, blood was collected in citrated tubes and was centrifuged for 10 minutes at 240 g at room temperature. Supernatant was diluted 1:50 with Tyrode buffer, then incubated for 30 minutes at room temperature in the dark with fluorochrome-labelled anti-human RPE-E-selectin (Southern Biotech, Birmingham, AL, USA) to detect EMPs or anti-mouse PE-IgG (Beckman Coulter, Marseille, France) serving as controls. During incubation, the cytometer was rinsed with FacsFlow™(BD Biosciences, Erembodegem-Aalst, Belgium). After fixation of the tubes with 300 μL of CellFix™(BD Biosciences, Erembodegem-Aalst, Belgium), samples were ready to be analyzed by flow cytometry. Microparticles were gated by a size of less than 1 μm using beads (Biocytex, Marseille, France) with a defined size of 0.9 μm (Figures 2a and b). Subsequently, microparticles positive for E-selectin (CD62E), which originated from activated endothelial cells were identified as activated EMPs (Figure 2c). Samples were analyzed at a flow rate of 12 μl/min for 180 seconds.

### Detection of EPCs by flow cytometry analysis

A 20 mL blood sample was used for enumeration of EPCs. Samples were collected in EDTA tubes and kept on ice at all times. Peripheral blood mononuclear cells were isolated by Ficoll density-gradient centrifugation at 4°C, resuspended in 1,000 μL of BSA 0.1% PBS and incubated for 10 minutes. After centrifugation at 1,000 rpm for five minutes at 4°C, the pellet was resuspended in 2 mL lysing buffer (constituting of NH_4_Cl, KHCO_3 _and tetrasodium-EDTA) and incubated for 10 minutes. Following a further centrifugation at 1,000 rpm for five minutes at 4°C and discarding supernatant, the pellet was washed twice in PBS and resuspended in 100 μL PBS. Thereafter, samples were incubated for 30 minutes in the dark with the following fluorochrome-labelled mAbs: 10 μL of anti-human FITC-CD34 (BD Biosciences, San Jose, CA, USA), anti-human PE-AC133 (Miltenyi Biotec GmbH, Auburn, CA, USA), and anti-human RPE-vascular endothelial growth factor-receptor 2 (VEGF-R2) (R&D Systems, Minneapolis, MN, USA), or anti-mouse FITC-/PE-IgG (Beckman Coulter, Marseille, France). Having washed the pellet twice with PBS, it was resuspended in 500 μL CellFix™(BD Biosciences, Erembodegem-Aalst, Belgium). Thereafter, tubes were ready for flow cytometry analysis. We measured 10,000 events in a lymphocyte gate. Triple-positive cells were identified by the expression of VEGF-R2 within the double-positive CD34+/CD133+ population gated. The number of EPCs was expressed as percentage of triple-positive cells of all lymphocytes.

### Measurement of vWF in resuscitated patients

In resuscitated patients, analysis was accompanied by photometric measurement of plasma vWF levels in the central laboratory of the university hospital Freiburg. Levels were compared with normal values of our institutional laboratory.

### Statistical analysis

Data are presented as means with the standard error of the mean and compared by Student's t-tests and Chi-Quadrate tests. Correlations between selected variables were estimated by Spearman's rank correlation coefficient. All tests were two-sided and a level of *P *less than 0.05 was accepted as significant. Data was analyzed with SPSS version 16.0 (SPSS Inc., Chicago, IL, USA).

## Results

### Baseline characteristics

In all resuscitated patients, overall duration of mechanical resuscitation varied from 5 to 120 minutes (mean 23.2 ± 2.7 minutes). The presenting initial rhythm was ventricular fibrillation or ventricular tachycardia in 58%, asystole or pulseless electrical activity in 42%. Fourty-two patients (76%) presented a presumable cardiac cause for cardiac arrest and 29 patients (53%) survived 10 days or more in the ICU (Table 1).

**Table 1:** Basic data of CPR and CAD patients

		CPR group			CAD group			*P* value
				
Group		CEC + EMP	EPC	All	CEC + EMP	EPC	All	
Number of patients		n = 40	n = 15	n = 55	n = 30	n = 9	n = 39	
**Age**	(years)	65.3 ± 2.2	66.8 ± 3.1	**65.7 ± 1.8**	64.9 ± 2.8	67 ± 4.3	**64.3 ± 2.3**	***0.32/ns***

**Gender**	Male	32 (80%)	10 (67%)	**42 (76%)**	26 (87%)	6 (67%)	**32 (82%)**	***0.63/ns***
	Female	8 (20%)	5 (33%)	**13 (24%)**	4 (13%)	3 (33%)	**7 (18%)**	

**Location of CPR**	in-hospital	13 (33%)	2 (13%)	**15 (27%)**				
	out-of-hospital	27 (67%)	13 (87%)	**40 (73%)**				

**Duration of CPR**	(min)	27.3 ± 3.5	12.3 ± 2	**23.2 ± 2.7**				

**Initial rhythm**	VF/VT	21 (52%)	11 (73%)	**32 (58%)**				
	Asystole/PEA	19 (48%)	4 (27%)	**23 (42%)**				

**Cause of cardiac arrest or hospital admission**	Cardiac	29 (72%)	13 (87%)	**42 (76%)**	30 (100%)	8 (89%)	**38 (97%)**	***0.11/ns***
	Non-cardiac	11 (28%)	3 (20%)	**14 (24%)**	0 (0%)	1 (11%)	**1 (3%)**	

**Coronary angiography**		27 (68%)	14 (93%)	**41 (75%)**	21 (70%)	6 (66%)	**27 (69%)**	***0.62/ns***

**Outcome**	Survival <10 days	21 (52%)	5 (33%)	**26 (47%)**	0 (0%)	0 (0%)	**0 (0%)**	
	Survival ≥ 10 days	19 (48%)	10 (67%)	**29 (53%)**	30 (100%)	9 (100%)	**39 (100%)**	

**Medication**	Vasopressors	32 (80%)	15 (100%)	**47 (85%)**	0 (0%)	0 (0%)	**0 (0%)**	
	Statins	14 (35%)	1 (7%)	**15 (27%)**	20 (67%)	3 (33%)	**23 (59%)**	***<0.05***

**Consecutive organ failure**	Acute heart failure	10 (25%)	3 (20%)	**13 (24%)**	0 (0%)	0 (0%)	**0 (0%)**	
	Acute renal failure	9 (23%)	1 (7%)	**10 (18%)**	0 (0%)	0 (0%)	**0 (0%)**	
	Acute liver failure	2 (1%)	0 (0%)	**2 (4%)**	0 (0%)	0 (0%)	**0 (0%)**	

**Co-morbidities**	CAD	26 (65%)	14 (93%)	**40 (73%)**	30 (100%)	7 (77%)	**37 (95%)**	***0.09/ns***
	PAD	6 (15%)	5 (33%)	**11 (20%)**	10 (33%)	1 (11%)	**11 (28%)**	***0.25/ns***
	Congestive heart failure	6 (15%)	1 (7%)	**7 (13%)**	6 (20%)	3 (33%)	**9 (23%)**	***0.10/ns***
	Pulmonary disease	12 (30%)	3 (20%)	**15 (27%)**	6 (20%)	4 (44%)	**10 (26%)**	***0.89/ns***
	Renal insufficiency	20 (50%)	6 (40%)	**26 (47%)**	8 (27%)	4 (44%)	**12 (31%)**	***0.07/ns***
	Liver insufficiency	7 (18%)	1 (7%)	**8 (15%)**	3 (10%)	0 (0%)	**3 (8%)**	***0.14/ns***

**Cardiovascular risk factors**	Hypertension	22 (55%)	4 (27%)	**26 (47%)**	17 (57%)	4 (44%)	**21 (54%)**	***0.49/ns***
	Diabetes	11 (28%)	5 (33%)	**16 (29%)**	3 (10%)	1 (11%)	**4 (10%)**	***<0.05***
	Hyperlipidemia	8 (20%)	1 (7%)	**9 (16%)**	15 (50%)	2 (22%)	**17 (44%)**	***<0.01***
	Smoking	8 (20%)	5 (33%)	**13 (24%)**	10 (33%)	4 (44%)	**14 (36%)**	***0.12/ns***

Basic data of the resuscitation group, including initial rhythm, outcome, location, and duration of CPR, and comparison of clinical variables in the CPR and CAD group. Both groups are comparable in baseline variables including age, gender, probable cause of cardiac arrest/cause of hospital admission, incidence of CAD, coronary angiography, congestive heart failure, and other relevant secondary disorders. There are slight but significant differences in distribution of cardiovascular risk factors as well as co-medication with statins.

CAD, coronary artery disease; CEC, circulating endothelial cells; CPR, cardiopulmonary resuscitation; EMP, endothelial microparticles; EPC, endothelial progenitor cells; ns, not significant; PAD, peripheral artery disease; PEA, pulseless electrical activity; VF, ventricular fibrillation; VT, ventricular tachycardia.

As more of the CPR patients in the smaller EPC study population presented ventricular fibrillation or ventricular tachycardia as the initial rhythm compared with patients in the CEC and EMP study (87% vs. 67%), duration of CPR in the EPC study group was shorter (27.3 ± 3.5 minutes vs. 12.3 ± 2.0 minutes), and outcome was better (survival ≥10 days in 67% vs. 48% of patients). Patients in the EPC study were showing higher rates of out-of-hospital cardiac arrests (87% vs. 67%), and a lower incidence of acute renal failure (7% vs. 23%) compared with resuscitated patients in the CEC and EMP study (Table 1).

Average time from ROSC to blood sampling was 2 hours 48 minutes ± 17 minutes in the CEC and EMP study. The second blood sample was collected 26 hours 45 minutes ± 1 hour 15 minutes after ROSC. Blood samples in the EPC study were collected on the second day after ROSC.

Comparing all patients in both studies, patients of the resuscitation and CAD group were comparable in baseline characteristics such as gender and age at time of investigation (65.7 ± 1.8 years in the resuscitation group vs. 64.3 ± 2.3 years in the control group; *P *= 0.32 not significant (ns)). Of the resuscitated patients, 73% were presenting significant CAD versus 95% of the control group. Most of the patients in both groups underwent coronary angiography (75% in the resuscitation group and 69% in the CAD group). There were differences in the cardiovascular risk profile of the two groups: CAD patients had a higher incidence of hyperlipidemia (16% vs. 44%; *P *< 0.05) and a trend to a higher prevalence of CAD and, hence, more of them were treated with statins (27% vs. 59%; *P *< 0.01). The groups had a comparable profile of secondary disorders including pulmonary disease, and renal and liver insufficiency (Table 1).

All measurements were also performed in healthy controls (nine in the CEC and EMP study and five in the EPC study), taking no medication and carrying no cardiovascular risk. Age at time point of investigation in the two groups was 30.5 ± 1.1 years and 37 ± 7 years, respectively.

### Detection of CECs by flow-cytometry analysis and correlation with duration of CPR

**Figure 3:**
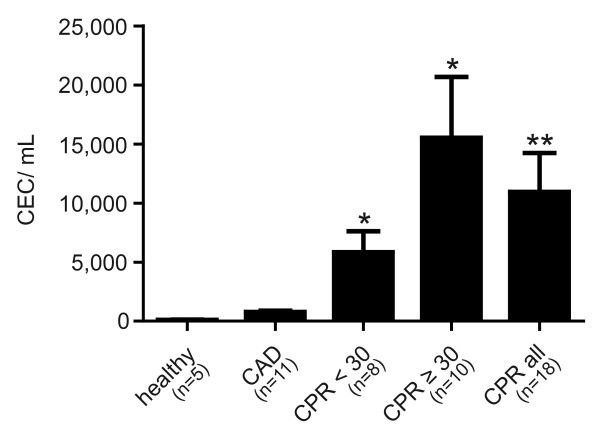
**Elevation of circulating endothelial cells in patients after CPR**. Levels of circulating endothelial cells (CEC) in peripheral blood obtained from healthy subjects, patients with coronary artery disease (CAD) and after cardiopulmonary resuscitation (CPR) for less than 30 minutes (CPR <30 min) and longer than 30 minutes (CPR ≥30 min), and in all resuscitated patients (CPR all). The number of CEC in all resuscitated patients was significantly higher compared with those in both control groups. *** *P *< 0.0005 versus control; ** *P *< 0.005 versus control.

**Figure 4:**
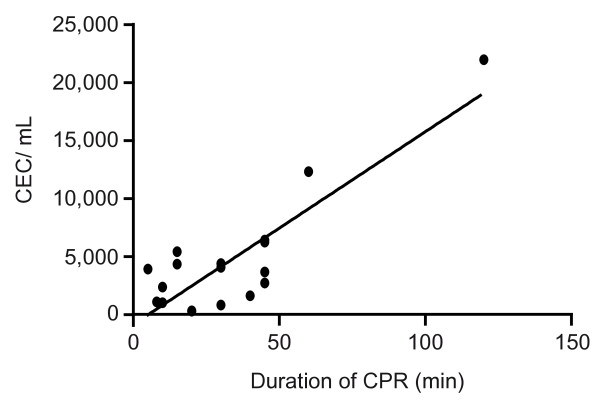
**Positive correlation of CEC count with duration of CPR**. Correlation between circulating endothelial cell (CEC) counts and the duration of cardiopulmonary resuscitation (CPR) showing a significant positive correlation. The rising numbers of CEC in peripheral blood with longer duration of resuscitation suggest a greater extent of endothelial damage during ongoing CPR (correlation coefficient 0.84; *P *< 0.01).

After CPR, we found a highly increased number of CECs in resuscitated patients. The mean number of CECs was 4,494.1 ± 1,246 cells/mL in patients after CPR. The number of CECs in resuscitated patients was significantly higher than in patients with stable CAD (mean number 312.7 ± 41 cells/mL; *P *< 0.005) and in healthy controls (mean number 47.5 ± 3.7 cells/mL; *P *< 0.0005; Figure 3). Patients who underwent prolonged CPR of 30 minutes or longer showed substantially elevated levels of circulating endothelial cells (mean number 6,216.6 ± 2,057 cells/mL) compared with patients resuscitated less than 30 minutes (mean number 2,340.9 ± 703.5 cells/mL; *P *= 0.13; ns). As depicted in Figure 4, CEC counts showed a significantly positive correlation with duration of CPR (R^2 ^= 0.84; *P *< 0.01; Figure 4).

### Detection of activated EMPs by flow cytometry analysis

**Figure 5:**
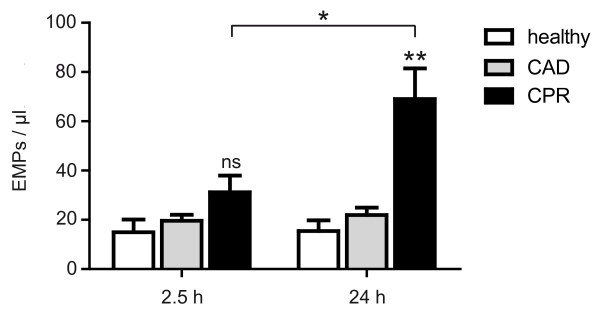
**Elevated endothelial microparticles in patients after CPR**. Levels of endothelial microparticles (EMPs) in peripheral blood obtained immediately after restoration of cardiopulmonary resuscitation (CPR; left) and 24 hours after return to spontaneous circulation (right) from healthy subjects (white bars), patients with coronary artery disease (CAD; grey bars) and after CPR (black bars). The number of EMPs in resuscitated patients immediately after return to spontaneous circulation was slightly higher compared with both control groups, showing a significant difference compared with controls 24 hours after return to spontaneous circulation). There is a significant rise in EMPs when comparing the two points in time after return to spontaneous circulation in the resuscitation group, reflecting an ongoing endothelial damage in the first 24 hours after CPR. *** *P *< 0.001 versus control; ** *P *< 0.005 versus control; * *P *< 0.01 versus control; ns, statistically not significant versus control.

As a second marker of endothelial injury, we investigated the presence of EMPs in the peripheral blood of resuscitated patients. Microparticles are characterized by size (that typically ranges from 0.1 to 1.0 μm) and exposure of antigens that reflect origin and activation state of the cells they originate from. Here, we chose E-selectin as a marker of endothelial cell activation. The mean number (31.2 ± 5.8 events/μL) was slightly increased immediately after CPR but did not differ significantly from CAD patients (mean number 19.7 ± 2.4 events/μL; *P *= 0.12) or healthy controls (mean number 15.0 ± 5.2 events/μL; *P *= 0.07). Twenty-four hours after ROSC, the mean numbers of EMPs (69.1 ± 12.4 events/μL) in resuscitated patients were significantly higher than in the CAD group (mean number 22.0 ± 3.0 events/μL; *P *< 0.005) and in healthy subjects 15.4 ± 4.4 events/μL; *P *< 0.001; Figure 5). In addition, in patients after successful resuscitation the number of EMPs increased significantly in the first 24 hours (*P *< 0.01). In controls, there was no significant difference between numbers of EMPs in a follow up of 24 hours (CAD: *P *= 0.55; healthy: *P *= 0.95).

Nine of the patients included, who were pre-treated with statins, immediately showed slightly lower EMP counts (22.3 ± 5.7 vs. 41.3 ± 12.6 events/μL; *P *= 0.23) and 24 hours after ROSC (62.6 ± 19.9 vs. 81.1 ± 19.2 events/μL; *P *= 0.49) compared with those without statin pre-treatment.

### Detection of EPC by flow cytometry analysis

**Figure 6:**
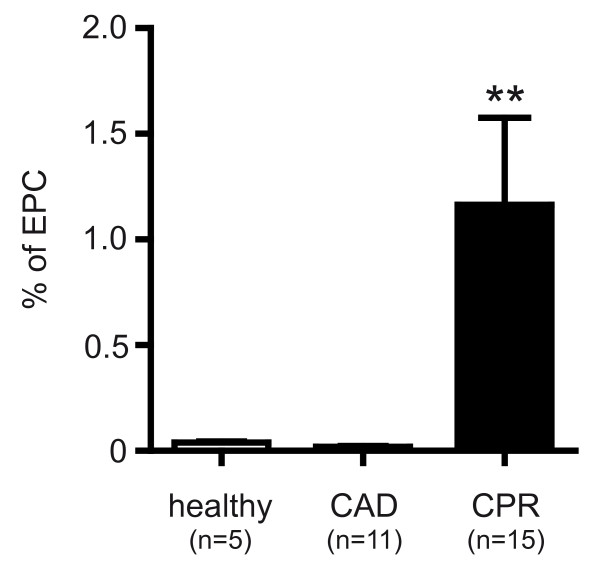
**Elevation of endothelial progenitor cells in patients after CPR**. Three-color flow cytometry evaluation of endothelial progenitor cells (EPCs) in healthy subjects (left), patients with coronary artery disease (CAD; middle) and after cardiopulmonary resuscitation (CPR; right). EPC count, expressed in percentage of gated lymphocytes, was significantly higher in resuscitated patients compared with both control groups, pointing to early onset of endothelial repair after CPR. ** *P *< 0.005 versus control.

To assess vascular repair following endothelial injury in patients after CPR, circulating EPCs were measured in peripheral blood of resuscitated patients in a follow-up study. Percentage of circulating EPCs in resuscitated patients were significantly higher than in control patients (1.16 ± 0.41% of gated lymphocytes in patients after CPR versus 0.02 ± 0.01% of gated lymphocytes (*P *< 0.005) in CAD and 0.04 ± 0.01% of gated lymphocytes (*P *< 0.005) in healthy controls; Figure 6). EPCs were identified by flow cytometry analysis as cells positive for CD34 (expressed on developmentally early stem and progenitor cells), CD133 (a marker expressed on immature cells) and VEGF-R2 (receptor mediating almost all of the known cellular responses to VEGF and essential for engraftment of hematopoietic stem and progenitor cells).

### vWF levels in resuscitated patients and correlation with other markers

**Figure 7:**
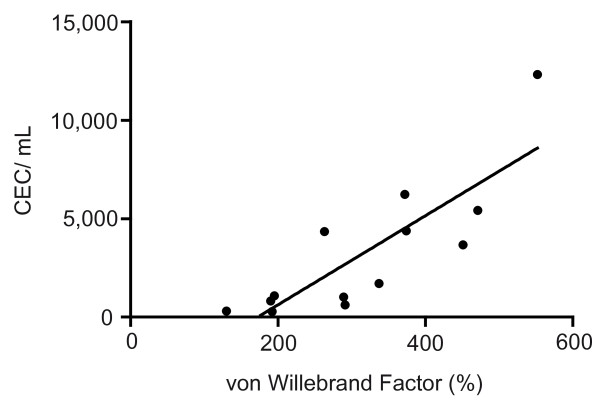
**Positive correlation of CEC count with von Willebrand factor**. Positive correlation between circulating endothelial cell (CEC) counts and von Willebrand factor levels in resuscitated patients, underlining the reliability of CEC count in detection of endothelial damage (correlation coefficient 0.77; *P *< 0.05).

Resuscitated patients showed significant elevated plasma levels of vWF immediately (296.5 ± 73.4%; *P *< 0.05) and 24 hours after ROSC (304.4 ± 22.8%; *P *< 0.001), compared with normal values of our institutional laboratory (50 to 160%), indicating endothelial damage or dysfunction. Furthermore, vWF levels in resuscitated patients correlated significantly with CEC count (R^2 ^= 0.77; *P *< 0.05; Figure 7).

## Discussion

The vascular endothelium lining the blood vessels is one of the most complex and sensible organ systems. Its integrity is an important precondition for a regular function of the circulatory system. The endothelium governs a host of fundamental physiologic functions: endothelial cells serve as signal transduction cells for a variety of chemical and mechanical stimuli, produce cytokines, or other paracrine signalling molecules, regulate leukocyte adhesion and migration, and maintain the balance between pro- and anticoagulant mechanisms [25,26].

The data presented in this report show the occurrence of severe endothelial damage in patients after successful CPR, and in a follow-up study, of a subsequent endothelial regeneration. In summary, we found a rapid and severe increase in CECs directly after CPR and a delayed increase in EMPs. This was paralleled by elevated levels of vWF, immediately and 24 hours after ROSC, compared with normal values. We chose vWF to support the flow cytometric measurements by a conventional and well-established marker of disturbed endothelium [13]. As levels of vWF showed a positive correlation with CEC count in resuscitated patients, these data underline the reliability of CEC count in the detection of endothelial damage. These results indicate early endothelial damage and ongoing endothelial dysfunction detected by elevated CECs and EMPs in resuscitated patients compared with control groups. Furthermore, numbers of EPCs increased on the second day after ROSC, which points to an early initiation of endothelial regeneration. The direct comparison with patients with stable CAD undergoing coronary intervention excluded effects possibly caused by CAD or coronary intervention.

In this study we could also demonstrate a significant positive correlation between the CEC count and the duration of CPR. In the literature, a long period of ischemia and longer duration of resuscitation efforts are associated with poor outcome [27,28], and the timing of the ischemic insult and the length of the reperfusion have been shown to correlate with endothelial dysfunction variables and biochemical or histological evidence for cellular damage [25,29]. Therefore, the correlation described in this study might reflect a greater extent of endothelial damage occurring during longer periods of CPR. However, we have to state that this positive correlation is largely due to a small number of patients with a long duration of CPR with CEC values outside the data cloud.

After cardiac arrest and mechanical resuscitation, there is growing evidence for an ischemia and reperfusion syndrome resulting in several inflammation cascades, including activation of leucocytes [30], up-regulation of selectins [4,6], and adhesion molecules [5,31] on the surface of the endothelium. Adrie and colleagues reported a 'sepsis-like syndrome' after resuscitation, with an increase in circulating interleukins, TNF, and a generalized systemic inflammatory response [3]. Researchers recently stated that an early toll-like receptor 4-induced vascular injury might be an important trigger of the systemic inflammatory response in resuscitated mice [32]. In analogy to the data obtained in our study, there are reports of elevated numbers of CECs in critically ill patients presenting with acute respiratory distress syndrome [33] and severe inflammatory disease conditions such as septic shock [34]. Hence, we excluded patients with septic shock from the study. The relatively few patients in the study with in-hospital arrests and the high percentage of patients with cardiac-related arrest suggest that most patients were not critically ill prior to cardiac arrest. Moreover, in critically ill patients, who did not undergo CPR, CEC counts were only slightly elevated (comparable with the levels of the CAD group; data not shown). Therefore, it seems to be unlikely that critical illness or ICU procedures themselves contribute to the elevation of CECs. The currently available data does not allow for a clear differentiation of whether the increase of CECs is the cause or effect of the inflammatory reaction taking place. The very early elevation of CECs to a vast extent after CPR might be an indicator that endothelial damage could be the initial step in post-resuscitation disease resulting in secondary inflammatory reactions. Conceivable triggers for endothelial damage might be hypoxia [30] or shear stress [35,36]. Therefore, it seems possible that shear stress during mechanical chest compression or the action itself is another cause of enhanced detachment of CEC from the endothelial layer.

We obtained comparatively high absolute values of CECs in this study, compared with the literature. In our opinion this indicates massive endothelial damage after CPR that largely exceeds the values detected in other diseases described so far in the available literature. This underlines the severity of this life-threatening condition, associated with complete discontinuation of circulation and high mortality. On the other hand, CEC counts in the literature vary from 15 to 670 cells/mL in various disease states [33,37], which points out the inhomogeneity of this relatively new method. Furthermore, different methodical approaches might change the absolute values.

Interestingly, and in contrast to the CECs, the number of EMPs in resuscitated patients rises further in the first 24 hours after ROSC, hence reflecting an ongoing process of endothelial damage. As EMPs are elevated in several systemic inflammatory diseases such as vasculitis [38] and sepsis [39], the noticeable increase in EMP numbers could be due to the systemic inflammatory response occurring after CPR maintaining endothelial injury. EMP may express adhesion molecules specific to mature endothelial cells, such as platelet-endothelial cell adhesion molecule-1 (CD31), VE-cadherin (CD144), or MCAM (CD146). Activation of endothelial cells with TNF-α induced the formation of EMPs [16] exposing adhesion-cell molecules, including E-selectin (CD62E) or intercellular adhesion molecule-1 (CD54). In this study, we measured activation-induced EMPs by detection of E-selectin-positive microparticles. A possible explanation for the ongoing endothelial injury in the post-resuscitation period could be ischemia and reperfusion during cardiac arrest and mechanical resuscitation.

Interestingly, patients treated with statins prior to cardiac arrest showed slightly lower EMP counts. These results indicate a potential protective effect of statins on the endothelium during and after ischemia and reperfusion and encourage further investigation of the effect of statin treatment in post-resuscitation care.

Finally, in a smaller population, we were able to detect elevated numbers of EPC in patients on the second day after CPR as an indicator of the early onset of endothelial repair. EPC-mediated vascular repair has been shown to be associated with normalization of endothelial function and restoration of blood flow at the site of injury [21]. These circulating cells are capable of endothelial differentiation and homing to ischemic tissues [20]. EPCs are considered to originate from hematopoietic stem cells, which are positive for CD34 and VEGF-R2 and immature marker protein CD133 [40]. CD34+ blood cell counts are widely used to obtain 'mobilized' hematopoietic stem/progenitor cells from peripheral blood [41]. Catecholamines are known to induce angiogenesis in tumor tissues [42] and dopamine has been shown to mobilize EPCs from the bone marrow during tumor growth [43]. Nevertheless, norepinephrine failed to increase CD34+ levels in heart failure patients [44]. In the EPC study group, all CPR patients received vasopressors such as norepinephrine and epinephrine but none of them received dopamine. Moreover, a small control group of critically ill patients at our ICU, who did not undergo CPR but received vasopressors and mechanical ventilation, showed no elevation of EPCs (data not shown). However, we cannot exclude the possibility of any influence of catecholamines or ICU procedures administered.

A limitation of the present study is its observatory design. Further experimental studies could allow a more detailed investigation of causes and effects of endothelial damage in post-resuscitation disease. Moreover, experimental studies would allow the assessment of possible therapeutic interventions. In the current study, the number of patients was rather small, particularly in the EPC study. However, the described effects are overwhelming and obtain clear statistical significance.

## Conclusions

In this study we provide evidence for an endothelial injury occurring in patients after CPR. The obtained data suggest a two-step process: The early stage during and directly after CPR is prevailed by severe endothelial damage. Within the following 24 hours, inflammation and endothelial repair are taking place. These results could be the basis for further interventional studies with the aim of developing new therapeutic and prognostic strategies in post-resuscitation care. As a perspective, endothelial protection (e.g. statins), anti-inflammatory drugs (e.g. corticosteroids), as well as regenerative therapies (e.g. growth factors) could be promising future therapeutic strategies in the early phases after CPR.

## Key messages

• Patients after successful CPR show an early and severe endothelial injury.

• Endothelial microparticles, as a sign of endothelial inflammation, rise within the first 24 hours after ROSC.

• On the second day after successful CPR, patients present elevated markers of endothelial repair.

## Abbreviations

BSA: bovine serum albumin; CAD: coronary artery disease; CEC: circulating endothelial cells; CPR: cardiopulmonary resuscitation; EMP: endothelial microparticles; EPC: endothelial progenitor cells; FITC: fluorescein isothiocyanate; MAb: monoclonal antibodies; MCAM: melanoma cell adhesion molecule; ns: not significant; PBS: phosphate-buffered saline; PCI: percutaneous coronary intervention; ROSC: return to spontaneous circulation; TNF: tumor necrosis factor; VEGF-R2: vascular endothelial growth factor-receptor 2; vWF: Von Willebrand factor.

## Competing interests

The authors declare that they have no competing interests.

## Authors' contributions

KF and MS were responsible for the conception and the design of the study, for the acquisition, analysis, and interpretation of data as well as for the writing of the manuscript. LF acquired data for CEC and EMP measurements. JS acquired data for EPC measurement. TS contributed to the writing of the manuscript and was responsible for revising it critically. NB acquired data. MO was responsible for statistical analysis of data. CM gave important advice for completion of the manuscript and revised it critically. CB contributed important intellectual content and gave final approval for the version to be published. HJB participated substantially in the conception of the study, analysis, and interpretation of data as well as in the writing of the manuscript. All authors read and approved the final manuscript.

## References

[B1] Negovsky VA The second step in resuscitation: The treatment of the "postresuscitation disease" Resuscitation 1972 1 1 7 10.1016/0300-9572(72)90058-5 4653025

[B2] Adrie C Laurent I Monchi M Cariou A Dhainaou JF Spaulding C Postresuscitation disease after cardiac arrest: a sepsis-like syndrome? Curr Opin Crit Care 2004 10 208 212 10.1097/01.ccx.0000126090.06275.fe 15166838

[B3] Adrie C Adib-Conquy M Laurent I Monchi M Vinsonneau C Fitting C Fraisse F Dinh-Xuan T Carli P Spaulding C Dhainaut JF Cavaillon JM Successful cardiopulmonary resuscitation after cardiac arrest as a "sepsis-like" syndrome Circulation 2002 106 562 568 10.1161/01.CIR.0000023891.80661.AD 12147537

[B4] Gando S Nanzaki S Morimoto Y Kobayashi S Kemmotsu O Alterations of soluble L- and P-selectins during cardiac arrest and CPR Intensive Care Med 1999 25 588 593 10.1007/s001340050907 10416910

[B5] Gando S Nanzaki S Morimoto Y Kobayashi S Kemmotsu O Out-of-hospital cardiac arrest increases soluble vascular endothelial adhesion molecules and neutrophil elastase associated with endothelial injury Intensive Care Med 2000 26 38 44 10.1007/s001340050009 10663278

[B6] Geppert A Zorn G Karth GD Haumer M Gwechenberger M Koller-Strametz J Heinz G Huber K Siostrzonek P Soluble selectins and the systemic inflammatory response syndrome after succesful cardiopulmonary resuscitation Crit Care Med 2000 28 2360 2365 10.1097/00003246-200007000-00030 10921565

[B7] Bridges AB McAlpine HM Pringle TH McLaren M Belch JJ Endothelial dysfunction in acute myocardial infarction after reperfusion Am Heart J 1993 126 451 445 10.1016/0002-8703(93)91066-N 8338019

[B8] Tsao PS Lefer AM Time course and mechanism of endothelial dysfunction in isolated ischemic- and hypoxic-perfused rat hearts Am J Physiol 1990 259 H1660 1666 2260693 10.1152/ajpheart.1990.259.6.H1660

[B9] Moroni G Del Papa N Moronetti LM Vitali C Maglione W Comina DP Urgnani F Sandri S Ponticelli C Cortelezzi A Increased levels of circulating endothelial cells in chronic periaortitis as a marker of active disease Kidney Int 2005 68 562 568 10.1111/j.1523-1755.2005.00434.x 16014033

[B10] Boos CJ Lip GYH Blann AD Circulating endothelial cells in cardiovascular disease J Am Coll Cardiol 2006 48 1538 1547 10.1016/j.jacc.2006.02.078 17045885

[B11] Mutin M Canavy I Blann A Bory M Sampol J Dignat-George F Direct evidence of endothelial injury in acute myocardial infarction and unstable angina by demonstration of circulating endothelial cells Blood 1999 93 2951 2958 10216090

[B12] Chong AY Blann AD Patel J Freestone B Hughes E Lip GYH Endothelial dysfunction and damage in congestive heart failure: relation of flow-mediated dilation to circulating endothelial cells, plasma indexes of endothelial damage, and brain natriuretic peptide Circulation 2004 110 1794 1798 10.1161/01.CIR.0000143073.60937.50 15364797

[B13] Sadler JE Biochemistry and genetics of von Willebrand factor Annu Rev Biochem 1998 67 395 424 10.1146/annurev.biochem.67.1.395 9759493

[B14] Geppert A Zorn G Delle-Karth G Koreny M Siostrzonek P Heinz G Huber K Plasma concentrations of von Willebrand factor and intracellular adhesion molecule-1 for prediction of outcome after successful cardiopulmonary resuscitation Crit Care Med 2003 31 805 811 10.1097/01.CCM.0000054861.69462.B5 12626988

[B15] Ahn YS Cell-derived microparticles: "Miniature envoys with many faces" J Thromb Haemost 2005 3 884 887 10.1111/j.1538-7836.2005.01347.x 15869581

[B16] Diamant M Tushuizen ME Sturk A Nieuwland R Cellular microparticles: new players in the field of vascular disease? Eur J Clin Invest 2004 34 392 401 10.1111/j.1365-2362.2004.01355.x 15200490

[B17] Bernal-Mizrachi L Jy W Jimerez JJ Pastor J Mauro LM Horstman LL de Marchena E Ahn YS High levels of circulating endothelial microparticles in patients with acute coronary syndromes Am Heart J 2003 145 962 970 10.1016/S0002-8703(03)00103-0 12796750

[B18] Piccin A Murphy WG Smith OP Circulating microparticles: pathophysiology and clinical implications Blood Rev 2007 21 157 171 10.1016/j.blre.2006.09.001 17118501

[B19] Urbich C Dimmeler S Endothelial progenitor cells: characterization and role in vascular biology Circ Res 2004 95 343 353 10.1161/01.RES.0000137877.89448.78 15321944

[B20] Asahara T Murohara T Sullivan A Silver M van der Zee R Li T Witzenbichler B Schatteman G Isner JM Isolation of putative progenitor endothelial cells for angiogenesis Science 1997 275 964 967 10.1126/science.275.5302.964 9020076

[B21] Hill JM Zalos G Halcox JPJ Schenke WH Waclawiw MA Quyyumi AA Finkel T Circulating endothelial progenitor cells, vascular function, and cardiovascular risk N Engl J Med 2003 348 593 600 10.1056/NEJMoa022287 12584367

[B22] Schmidt-Lucke C Rössig L Fichtlscherer S Vasa M Britten M Kämper U Dimmeler S Zeiher AM Reduced number of circulating endothelial progenitor cells predicts future cardiovascular events: proof of concept for the clinical importance of endogenous vascular repair Circulation 2005 111 2981 2987 10.1161/CIRCULATIONAHA.104.504340 15927972

[B23] Vasa M Fichtlscherer S Aicher A Adler K Urbich C Martin H Zeiher AM Dimmeler S Number and migratory activity of circulating endothelial progenitor cells inversely correlate with risk factors for coronary artery disease Circ Res 2001 89 E1 E7 10.1161/hh1301.093953 11440984

[B24] Goon PKY Lip GYH Boos CJ Stonelake PS Blann AD Circulating endothelial cells, endothelial progenitor cells, and endothelial microparticles in cancer Neoplasia 2006 8 79 88 10.1593/neo.05592 16611400 PMC1578513

[B25] Karimova A Pinsky DJ The endothelial response to oxygen deprivation: biology and clinical implications Intensive Care Med 2001 27 19 31 10.1007/s001340000790 11280633

[B26] Vinten-Johansen J Involvement of neutrophils in the pathogenesis of lethal myocardial reperfusion injury Cardiovasc Res 2004 61 481 497 10.1016/j.cardiores.2003.10.011 14962479

[B27] Ferguson RP Phelan T Haddad T Hinduja A Dubin NH Survival after in-hospital cardiopulmonary resuscitation South Med J 2008 101 1007 1011 18791505 10.1097/SMJ.0b013e318184ac77

[B28] Ishtiaq O Iqbal M Zubair M Qayyum R Adil M Outcome of cardiopulmonary resuscitation- predictors of survival J Coll Physicians Surg Pak 2008 18 3 7 18452659

[B29] Aliev G Obrenovich ME Seyidova D De La Torre JC Exploring ischemia-induced vascular lesions and potential pharmacological intervention strategies Histol Histopathol 2005 20 261 273 15578444 10.14670/HH-20.261

[B30] Böttiger BW Motsch J Braun V Martin E Kirschfink M Marked activation of complement and leukocytes and an increase in the concentrations of soluble endothelial adhesion molecules during cardiopulmonary resuscitation and early reperfusion after cardiac arrest in humans Crit Care Med 2002 30 2473 2480 10.1097/00003246-200211000-00012 12441757

[B31] Larmann J Schmidt C Gammelin H Van Aken HK Frenzel T Lanckohr C Lox M Boese N Jurk K Theilmeier G Intercellular adhesion molecule-1 inhibition attenuates neurological and hepatic damage after resuscitation in mice Anesthesiology 2005 103 1149 1155 10.1097/00000542-200512000-00008 16306726

[B32] Benhamou Y Favre J Musette P Renet S Thuillez C Richard V Tamion F Toll-like receptors 4 contribute to endothelial injury and inflammation in hemorrhagic shock in mice Crit Care Med 2009 37 1724 1728 10.1097/CCM.0b013e31819da805 19325486

[B33] He XL Liu Z Xia SY Vascular endothelial injuries and changes of blood coagulation and fibrinolysis indexes in patients with acute respiratory distress syndrome Chin Med Sci J 2004 19 252 256 15669181

[B34] Mutunga M Fulton B Bullock R Batchelor A Gascoigne A Gillespie JI Baudouin SV Circulating endothelial cells in patients with septic shock Am J Respir Crit Care Med 2001 163 195 200 11208646 10.1164/ajrccm.163.1.9912036

[B35] Cunningham KS Gotlieb AI The role of shear stress in the pathogenesis of atherosclerosis Lab Invest 2005 85 9 23 10.1038/labinvest.3700299 15568038

[B36] Inoguchi H Tanaka T Maehara Y Matsuda T The effect of gradually graded shear stress on the morphological integrity of a HUVEC-seeded compliant small-diameter vascular graft Biomaterials 2007 28 486 495 10.1016/j.biomaterials.2006.09.020 17034847

[B37] Woywodt A Kirsch T Haller H Haubitz M Circulating endothelial cells as a prognostic marker in thrombotic microangiopathy Am J Kidney Dis 2006 48 564 570 10.1053/j.ajkd.2006.06.013 16997052

[B38] Brogan PA Shah V Brachet C Harnden A Mant D Klein N Dillon MJ Endothelial and platelet microparticles in vasculitis of the young Arthritis Rheum 2004 50 927 936 10.1002/art.20199 15022336

[B39] Soriano AO Jy W Chirinos JA Valdivia MA Velasquez HS Jimenez JJ Horstman LL Kett DH Schein RM Ahn YS Levels of endothelial and platelet microparticles and their interactions with leukocytes negatively correlate with organ dysfunction and predict mortality in severe sepsis Crit Care Med 2005 33 2540 2546 10.1097/01.CCM.0000186414.86162.03 16276178

[B40] Rafii S Lyden D Therapeutic stem and progenitor cell transplantation for organ vascularization and regeneration Nat Med 2003 9 702 712 10.1038/nm0603-702 12778169

[B41] Sutherland DR Keating A Nayar R Anania S Stewart AK Sensitive detection and enumeration of CD34+ cells in peripheral and cord blood by flow cytometry Exp Hematol 1994 22 1003 1010 7522181

[B42] Lutgendorf SK Cole S Costanzo E Bradley S Coffin J Jabbari S Rainwater K Ritchie JM Yang M Sood AK Stress-related mediators stimulate vascular endothelial growth factor secretion by two ovarian cancer cell lines Clin Cancer Res 2003 9 4514 4521 14555525

[B43] Chakroborty D Chowdhury UR Sarkar C Baral R Dasgupta PS Basu S Dopamine regulates endothelial progenitor cell mobilization from mouse bone marrow in tumor vascularization J Clin Invest 2008 118 1380 1389 10.1172/JCI33125 18340382 PMC2267013

[B44] Carvalho VO Ruiz MA Bocchi EA Carvalho VO Guimarães GV Correlation between CD34+ and exercise capacity, functional class, quality of life and norepinephrine in heart failure patients Cardiol J 2009 16 426 431 19753521

